# In silico design of an epitope-based vaccine against PspC in *Streptococcus pneumoniae* using reverse vaccinology

**DOI:** 10.1186/s43141-023-00604-8

**Published:** 2023-12-12

**Authors:** Md. Nahian, Muhammad Shahab, Lincon Mazumder, Jonas Ivan Nobre Oliveira, Tanjina Akhtar Banu, Murshed Hasan Sarkar, Barna Goswami, Ahashan Habib, Shamima Begum, Shahina Akter

**Affiliations:** 1 Department of Microbiology, Jagannath University, Dhaka 1100, Bangladesh; 2 State key laboratories of chemical Resources Engineering. Beijing University of Chemical Technology, Beijing, 100029 China; 3 Department of Biology, Indiana State University, Terre Haute, United States; 4 Departamento de Biof ´ısica e Farmacologia, Universidade Federal do Rio Grande doNorte, 59072-970, Natal, RN, Brazil; 5 Bangladesh Council of Scientific and Industrial Research (BCSIR), Dhaka-1205, Bangladesh

**Keywords:** Epitope, Vaccine, B cell, T cell, Immunoinformatics, Reverse vaccinology

## Abstract

**Background:**

*Streptococcus pneumoniae* is a major pathogen that poses a significant hazard to global health, causing a variety of infections including pneumonia, meningitis, and sepsis. The emergence of antibiotic-resistant strains has increased the difficulty of conventional antibiotic treatment, highlighting the need for alternative therapies such as multi-epitope vaccines. In this study, immunoinformatics algorithms were used to identify potential vaccine candidates based on the extracellular immunogenic protein Pneumococcal surface protein C (PspC).

**Method:**

The protein sequence of PspC was retrieved from NCBI for the development of the multi-epitope vaccine (MEV), and potential B cell and T cell epitopes were identified. Linkers including EAAAK, AAY, and CPGPG were used to connect the epitopes. Through molecular docking, molecular dynamics, and immunological simulation, the affinity between MEV and Toll-like receptors was determined. After cloning the MEV construct into the PET28a ( +) vector, SnapGene was used to achieve expression in *Escherichia coli*.

**Result:**

The constructed MEV was discovered to be stable, non-allergenic, and antigenic. Microscopic interactions between ligand and receptor are confirmed by molecular docking and molecular dynamics simulation. The use of an in-silico cloning approach guarantees the optimal expression and translation efficiency of the vaccine within an expression vector.

**Conclusion:**

Our study demonstrates the potential of in silico approaches for designing effective multi-epitope vaccines against *S. pneumoniae*. The designated vaccine exhibits the required physicochemical, structural, and immunological characteristics of a successful vaccine against SPN. However, laboratory validation is required to confirm the safety and immunogenicity of the proposed vaccine design.

## Background

Pneumonia is a respiratory infection that specifically affects one or both lungs. The lungs are comprised of small sacs called alveoli, which are essential for the breathing process. However, in cases of persistent pneumonia, these alveoli can become filled with pus and fluid, causing painful breathing and reduced oxygen intake. As a result, it can lead to the development of acute respiratory syndrome [1]. *Streptococcus pneumoniae*, also known as pneumococcus, a bacterium that is gram-positive and non-motile, is the main cause of community-acquired pneumonia. Pneumococcal pneumonia predominantly affects infants below the age of 2, older adults, and people with weakened immune systems [2]. Pneumococcal invasive diseases comprise various illnesses such as invasive pneumococcal disease (IPD), bacteremia, sepsis, meningitis, empyema, sinusitis, and middle ear otitis media [2, 3]. As a consequence, almost 14 million cases of pneumococcal disease have been reported around the world, with an estimated 1.6 million fatalities owing to this infection annually [4]. The incidence of pneumonia among children in undeveloped nations was around 0.29 cases per person per year, whereas it was just 0.05 cases per person per year in developed nations [4, 5]. Hence, it is the top cause of mortality among children worldwide.

Research conducted in the USA in 2008 showed that pneumococci had evolved resistance to many kinds of antibiotics. These include quinolones, penicillin, macrolides, and cephalosporins [6]. Evidence from China indicates that *S. pneumoniae* is very resistant to many antibiotics, with rates of resistance of 95.8% to clindamycin, 95.2% to erythromycin, 93.6% to tetracycline, and 66.7% to trimethoprim/sulfamethoxazole [7]. Furthermore, the existing pneumococcal vaccines do not provide full protection against all strains of *Streptococcus pneumoniae* since there are more than 100 different serotypes of this bacterium. At present, there are two categories of pneumococcal vaccines: plain polysaccharide vaccines (PPV) and protein-conjugated polysaccharide vaccines (PCV) [8]. Due to its reliance on T cell-independent polysaccharide antigens, PPV does not effectively protect babies younger than two, who are at the highest risk for serious pneumococcal infections. On the other hand, PCV provides protection to children, but it has drawbacks such as a high cost, being difficult to manufacture, requiring multiple injections, and needing to be refrigerated. Additionally, PCV only covers certain pneumococcal serotypes found in developed countries [9, 10]. Both vaccines are limited to specific serotypes and can only induce immunity against those specific serotypes [9]. To address this limitation and protect against a wider range of *S. pneumoniae* serotypes, there is an urgent need for a vaccination approach that is not limited to specific serotypes.

An effective approach for vaccination against *Streptococcus pneumoniae* is to use an epitope-based strategy that targets specific epitopes using a group of conserved pneumococcal protein antigens. This method can serve as a feasible alternative to vaccines that are designed solely for specific serotypes [11]. By focusing on conserved protein antigens, epitope-based vaccines have the potential to provide broad protection against various strains or serotypes of pneumococcus [12, 13]. Over the years, researchers have extensively studied different pneumococcal proteins with the aim of creating a vaccine that can protect against pneumococcal diseases. These include pneumococcal surface proteins A and C (PspA/C) [14, 15], pneumococcal histidine triad (Pht) family members A, B, D, and E (PhtA, B, D, and E) [16, 17], and ATP binding cassette (ABC) transporters PsaA, PiuA, and PiaA [18, 19]. Among these antigens, PspC is one of the most attractive candidates because it is highly prevalent and highly conserved among *Streptococcus pneumoniae* strains [20]. PspC is a surface-exposed protein that can bind to human complement factor H and secretory immunoglobulin A, thereby evading the innate and adaptive immune systems. PspC also acts as an adhesin that mediates the attachment of the pneumococcus to epithelial cells and facilitates colonization and invasion [21]. These properties make PspC a promising target for the development of a vaccine that can provide broader protection against *S. pneumoniae.*

Recent advancements in immunoinformatics have revolutionized the development of epitope vaccines, offering cost-effective and time-efficient approaches along with an expanded range of vaccine design tools [22–24]. In recent years, scientists have extensively relied on computational approaches for selecting efficient epitopes and developing innovative vaccinations against a wide variety of diseases, including SARS-CoV-2 [25], *Burkholderia pseudomallei* [26], *Mycobacterium tuberculosis* [27], and *Staphylococcus aureus* [28]. Given the practicality and advantages of immunoinformatics-based vaccine design, the primary objective of this study is to progress the creation of a multi-epitope vaccine to safeguard against *Streptococcus pneumoniae* by specifically targeting the PspC protein. To create the vaccine, multiple B and T cell epitopes will be included, which were produced by utilizing various in silico methods. Vaccine structure were modeled and docked with TLR4 to obtain an effective immune response. In addition, molecular dynamics simulations were employed to validate the bound complex and investigate its properties. Lastly, the process of in silico cloning and codon optimization was conducted to evaluate the expression of chimeric proteins in a suitable host. The main strength of this study is the inclusion of more potent antigenic epitope-rich proteins in the vaccine, which can elicit diverse and robust immune responses. The significance of this approach has already been demonstrated in combating diseases like malaria [29], multiple sclerosis [30], and tumors [31].

## Methods

**Fig. 1:**
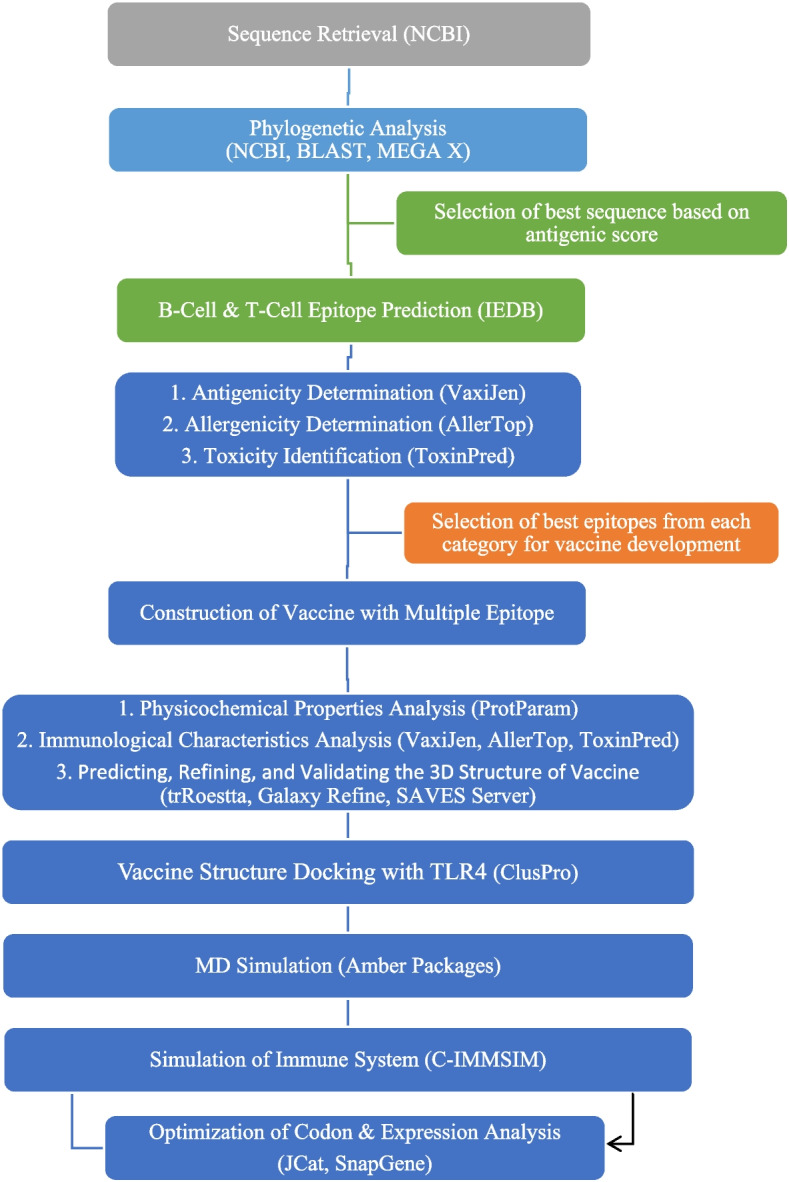
The procedure adopted in this research to develop the pneumococcal epitope-based vaccination

Figure 1 illustrates the complete workflow of this scientific study. The figure also displays the diverse set of tools utilized in this approach.

### Retrieval and initial analysis of protein sequences

The research began by acquiring the protein sequence of PspC from NCBI (https://www.ncbi.nlm.nih.gov/protein). Afterward, this sequence was submitted to protein–protein blast (Blastp) [32]. MUSCLE v3.6 program [33] was used to perform the multiple sequence alignment. Mega X was utilized to determine the evolutionary relationships between the sequences [34]. Subsequently, the antigenic characteristics of each protein sequence have been analyzed through the online tool VaxiJen version 2.0 [35], accessible at http://www.ddg-pharmfac.net/vaxijen/VaxiJen/VaxiJen.html. Subsequently, an allergic prediction scan was conducted on the protein sequences utilizing the AllerTOP v. 2.0 [36] web tool, accessible at https://www.ddg-pharmfac.net/AllerTOP/.

### Analysis of physical and chemical properties

ExPasy ProtParam was used to investigate the protein's physical and chemical characteristics [37], accessible at https://www.expasy.org/resources/protparam/. The purpose of this web tool is to provide a comprehensive analysis of the protein sequence by calculating various physical and chemical parameters. These parameters provide insights into the protein’s structure, function, stability, and interactions. For instance, the molecular weight reflects the protein’s size and complexity, the half-life estimates the protein’s longevity and degradation rate, and the GRAVY measures the protein’s hydrophobicity or hydrophilicity. These parameters help us determine the protein’s suitability for further analysis and application [38].

### B cell epitopes prediction

The identification of B-cell epitopes (BCEs), which are responsible for the formation of antibodies that confer humoral immunity, is an essential initial step in the construction of an epitope-based vaccine. Therefore, the IEDB Linear Epitope Prediction Tool v2 (http://tool.iedb.org/bcell/) [39] was used to make predictions about B cell epitopes. This tool uses a combination of amino acid scales and hidden Markov models to score the epitope potential of each residue in a protein sequence. The tool also incorporates solvent-accessible surface area calculations, as well as contact distances into its prediction of B cell epitope potential [40]. To ensure accuracy, the final selection of B cell epitopes involved a meticulous screening process using VaxiJen v2.0, AllerTOP v.2.0, and ToxinPred servers.

### Prediction of the MHC-specific epitopes

Using the IEDB website (http://tool.iedb.org/main/), T cell epitopes that trigger an effective immune response were analyzed by utilizing the commonly occurring fragment with the help of the MHC-1 (http://tools.iedb.org/mhci/) and MHC-2 tools (http://tools.iedb.org/mhcii/). To identify MHC-I restricted epitopes, the ANN 4.0 algorithm [41] was utilized, and for MHC-II restricted epitopes, NN-align 2 [42] was used. Then the epitopes were ranked according to their VaxiJen score, allergenicity, and toxicity.

### Determination of population coverage

Between populations of various ancestries, there are major differences in the expression and distribution of HLA alleles [43]. In this work, we estimated the proportion of the population that would be covered by MHC-I and MHC-II epitopes using the IEDB Analysis Resource, which can be found at http://tools.iedb.org/population/, to make an estimation of the percentage of the population that would be covered by our selected epitopes.

### Finalizing the construct

The most effective B and T cell epitopes have been combined sequentially using an appropriate linker to create a multi-subunit vaccine. In the creation of vaccines, linkers such as AAY, EAAAK, and CPGPG were used. Since the peptides used to make vaccines are not highly immunogenic, adjuvants must be used to elicit an immune response [44]. To facilitate purification experiments, the construct was modified by adding a hexa-histidine tail, also known as a poly-histidine tag.

### Physicochemical and immunological property analysis

The vaccine's physicochemical characteristics were determined using ExPasy of the ProtParam (https://web.expasy.org/protparam/) [37]. This included calculating its isoelectric pH, instability, aliphatic index, molecular weight, in vitro and in vivo half-life, as well as its GRAVY index. To evaluate the immunological properties of the vaccine, including antigenicity, allergenicity, and toxigenicity, comprehensive assessments were conducted using VaxiJen (https://www.ddgpharmfac.net/vaxijen/VaxiJen/VaxiJen.html) [35], AllerTOP (https://webs.iiitd.edu.in/raghava/toxinpred/multi_submit.php) [36], and ToxinPred (https://webs.iiitd.edu.in/raghava/toxinpred/multi_submit.php) [45].

### Predicting, refining and validating vaccine 3D structure

To estimate the 3D structure of our build sequence, we utilized the popular and publicly accessible trRosetta online tool (http://yanglab.nankai.edu.cn/trRosetta/) [46], a widely used and freely available tool for quick and accurate protein structure prediction. It constructs protein structure by using constrained Rosetta and direct energy minimizations, with the help of a deep neural network to determine the locations and orientations of the residues. GalaxyRefine [47] was utilized to refine the design of the vaccine and its receptor, which is accessible at http://galaxy.seoklab.org/. Five high-performing models were evaluated, and the one with the biggest increase in RAMA score was chosen. The RAMPAGE server, which is accessible at https://www.ccp4.ac.uk/html/rampage.html [48] was utilized to validate the vaccination model and make predictions about the Ramachandran plot. The Ramachandran plot showcases the representation of individual amino acids in relation to their F and Y values. The top-right quadrant of the plot is indicative of a greater probability of amino acids forming left-handed alpha helices, while the lower-left quadrant suggests right-handed alpha helices. Amino acids are represented by several ß-sheet types in the upper-left quadrant, such as twisted, parallel, and anti-parallel strands [48]. The darker sections indicate the amino acids that are highly favored, while the lighter regions signify that the amino acids are acceptable. On the other hand, the white regions suggest that the amino acids are either low in quality or strictly prohibited.

### Vaccine structure docking with TLR4

The vaccination has to engage the immune cell receptor in order to stimulate the immune system effectively. Therefore, we conducted docking research to evaluate the interaction between the constructed MEV and TLR4. The crystal structure of TLR4, identified by its PDB ID of 4G8A, was obtained through the protein data library at RCSB. The vaccine structure and TLR4 receptor were docked using Cluspro2.0, a publicly accessible web server, which can be accessed at https://cluspro.org/tutdock.php. [49]. It is a web-based program that performs protein–protein docking, to predict the three-dimensional structure of protein complexes based on interaction energies and centers. It is a reliable and widely used tool that can compete with the best human predictor groups in the CAPRI (Critical Assessment of Predicted Interactions) experiment, which is a community-wide blind test of protein–protein docking methods [50].

### MD simulation

Molecular dynamics studies serve a critical part in any in silico investigation by evaluating the stability of the protein–protein complex [51]. We utilized MD simulations using the Amber 22 setup to investigate the TLR4-vaccine complex's stability. This software allows the exploration of the structural aspects, dynamic behaviors, and molecular interactions inherent to biomolecules across various environmental contexts and under diverse conditions. It facilitates accelerated simulations through the utilization of parallel central processing unit (CPU) or graphics processing unit (GPU) hardware, while also offering access to advanced force fields and computational methods to enhance the level of analysis and accuracy of simulations [52].

### Immune simulation

The C-ImmSim server (https://kraken.iac.rm.cnr.it/C-IMMSIM/) was utilized to simulate the immune system computationally in order to validate the immunological response and immunogenic profile of the constructed design [53]. This online server predicts the immune response in a manner similar to the innate response of the body.

### Expression analysis

Using the Java Codon Adaptation Tool (JCat) server (http://www.jcat.de/) [54], the designed vaccine was reverse-translated and codon-optimized, which facilitated its expression in an *E. coli* expression system. JCat includes three additional factors: the locations of restriction enzyme cleavage, the sites where the bacterial ribosome binds, and the sites where transcription is terminated independently of Rho. JCat has provided indications of protein expression levels in the form of percentages of CG content and the codon adaptation index (CAI). The CAI must be greater than 0.8 and less than or equal to 1.0, and the CG percentage must be between 30 and 70%. Using SnapGene (https://www.snapgene.com/), the generated sequence was then inserted into pET-28a ( +) to ensure the expression of the vaccine [55].

## Results

### Retrieval of sequences, phylogenetic categorization and sequence prioritization

**Fig. 2:**
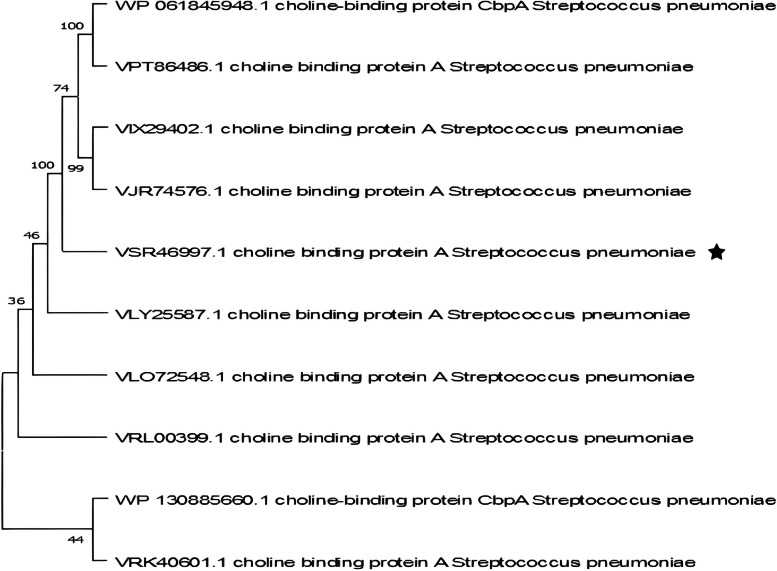
Phylogenetic tree illustrating the relationship among the top 10 sequences, including our reference protein sequence (marked with a ★ symbol). These sequences were identified using the BlastP search algorithm in a non-redundant database. The Poisson correction method was utilized to compute the evolutionary distances, and these distances are conveyed through the average number of alterations in amino acids per location

The PspC reference sequence was obtained from the NCBI database (accession number VSR46997.1). Using Blastp, the top 10 sequences were extracted, and then multiple sequence alignment was performed using the MUSCLE v3.6 software. As shown in Fig. 2, a phylogenetic tree was constructed in the software MEGA X in order to visualize the evolutionary relationships between the sequences. Amongst the compared sequences, the protein sequence obtained from NCBI (accession number VSR46997.1) exhibited the highest antigenic potency or immunogenicity, with a notable VaxiJen score of 0.8245. In addition, the Aller-TOP server concurred that no allergens were present in this sequence. The combined use of VaxiJen and AllerTOP confirms this protein's viability as a vaccine target.

### Physiochemical characterization

With the use of the ExPasy Protparam program, we were able to determine that this sequence consists of 251 amino acids and has a molecular weight of 28,465.62 [37]. The pI value was 8.52. The computed value of the instability index is 38.17, which indicates the protein is stable. Aliphatic index of 55.82 determined that our protein is a stable one, along with temperature assortment [56]. The C_3465_H_5437_N_963_O_1116_S_13_ formula identified the number of sulfur (S), oxygen (O), nitrogen (N), carbon (C), and hydrogen (H). The value of GRAVY was − 1.083.

### Anticipating B cell epitopes

The prevention of microbial infections is largely dependent on the presence of B-cell epitopes. These epitopes possess modified traits that guide B cells in identifying and triggering diverse immune responses that facilitate the recognition of particular microbial infections. Using the IEBD analysis tool, 23 linear B cell epitopes were computed with a cutoff score of 0.400. Allergenic and poisonous epitopes were eliminated, and only antigenic, non-allergic, and non-toxic epitopes were chosen. On average, 12 out of 23 B cell epitopes were considered effective. As shown in Table 1, antigenicity ranged from a maximum of 1.1522 to a minimum of 0.1247, with an average of 0.66. The threshold value for antigenic determination of this protein was 0.4, so any number greater than that may be regarded as an antigenic determinant. Twelve antigenic epitopes were selected in the end.

**Table 1:** Using the Kolaskar and Tongaonkar antigenicity approach [57], B-cell epitopes were anticipated with the help of the IEDB B-cell epitope prediction program

Start	End	Peptide	Length	Antigenicity	Allergenicity	Toxicity
510	515	WYYLNA	6	1.1522	Non-allergen	Non-toxin
282	297	LPSPSLKLGKKVAEAE	16	1.0333	Non-allergen	Non-toxin
650	656	WYYLEAS	7	1.0143	Non-allergen	Non-toxin
69	75	NKKLQLD	7	0.7865	Non-allergen	Non-toxin
109	125	AELPSKIKAKLDAAFDQ	17	0.743	Non-allergen	Non-toxin
404	414	EQPQPAPAPQP	11	0.6585	Non-allergen	Non-toxin
7	38	ERKVHYSIRKFSVGVAS VVVASLVMGSVVHAT	32	0.6142	Non-allergen	Non-toxin
569	575	SWYYLNA	7	0.6079	Non-allergen	Non-toxin
549	554	SWYYLN	6	0.5103	Non-allergen	Non-toxin
529	535	SWYYLNS	7	0.3833	Non-allergen	Non-toxin
663	685	QWFKVSDKWYYVNGLGA LAVNTT	23	0.3554	Non-allergen	Non-toxin
97	105	LRELNVLED	9	0.1247	Non-allergen	Non-toxin

### Anticipating T cell epitopes

Numerous data entries with IC50 values ranging from 2.86 to 48,867.22 were generated by the calculations of MHC-I and MHC-II-restricted epitopes. These entries were further analyzed, and potential epitopes were identified by filtering them based on an IC50 value of 250 or less [58]. A low IC50 value indicates that the vaccine epitope can be active at sub-lethal doses, leading to reduced systemic toxicity after administration. This means that the vaccine's epitopes can produce a strong immune response with a smaller amount of the vaccine [59].

### MHC-I-restricting epitopes

Table 2 lists the eight epitopes selected as target epitopes from a pool of 450 anticipated MHC-I epitopes. The IC50 values (below 250) of these epitopes were used as selection criteria, along with their high antigenic potential and lack of allergenicity and toxicity.

**Table 2:** Epitopes that are restricted by MHC-I and have been predicted using IEDB

**No.**	**length**	**Peptide**	**All** allel**es**	**Antigenicity**	**Allergenicity**	**Toxicity**
1	9	KVSDKWYYV	HLA-A*02:06,HLA-A*02:01,HLA-A*02:03,HLA-A*30:01,HLA-A*31:01,HLA-A*68:02,HLA-A*32:01	1.2339	Non-allergen	Non-Toxin
2	9	RNYPTNTYK	HLA-A*30:01,HLA-A*31:01,HLA-A*03:01,HLA-A*11:01	0.6313	Non-allergen	Non-toxin
3	9	TLIIKLSAI	HLA-A*02:03,HLA-B*08:01,HLA-A*02:06	0.4851	Non-allergen	Non-toxin
4	10	SSNMAKTEYR	HLA-A*31:01,HLA-A*68:01,HLA-A*11:01	1.3595	Non-allergen	Non-toxin
5	9	SNMAKTEYR	HLA-A*31:01,HLA-A*68:01,HLA-A*33:01	1.1598	Non-allergen	Non-toxin
6	10	NANGAMATGW	HLA-B*58:01,HLA-B*57:01,HLA-B*53:01	0.7305	Non-allergen	Non-toxin
7	10	QVATSSNMAK	HLA-A*11:01,HLA-A*68:01,HLA-A*03:01	0.7747	Non-allergen	Non-toxin
8	9	LIIKLSAIK	HLA-A*68:01,HLA-A*11:01,HLA-A*03:01	0.641	Non-allergen	Non-toxin
9	10	TLIIKLSAIK	HLA-A*68:01,HLA-A*11:01,HLA-A*03:01	0.485	Non-allergen	Non-toxin

### MHC-II-restricting epitopes

Table 3 displays the 15 MHC-II epitopes with the lowest IC50 values among the 586 total. While selecting these epitopes, we only kept the ones that were antigenic, non-allergenic, and non-toxic and discarded the ones that did not meet these criteria.

**Table 3:** IEDB-predicted MHC-II-restricted epitope

**No.**	Peptide	**All** alleles	**Antigenicity**	**Allergenicity**	**Toxicity**
1	SWYYLNANGAMATGW	HLA-DRB1*01:01,HLA-DRB3*02:02,HLA-DRB1*13:02,HLA-DRB1*09:01,HLA-DRB1*04:01,HLA-DRB1*11:01,HLA-DQA1*05:01/DQB1*03:01,HLA-DQA1*01:02/DQB1*06:02,HLA-DRB5*01:01,HLA-DRB3*01:01,HLA-DRB1*04:05,HLA-DRB1*07:01	0.6068	Non-allergen	Non-toxin
2	SWYYLNASGAMATGW	HLA-DRB1*01:01,HLA-DRB3*02:02,HLA-DRB1*09:01,HLA-DRB1*11:01,HLA-DQA1*05:01/DQB1*03:01,HLA-DQA1*01:02/DQB1*06:02,HLA-DRB1*07:01,HLA-DRB1*13:02,HLA-DRB3*01:01,HLA-DRB1*04:01,HLA-DRB5*01:01,HLA-DRB1*04:05	0.502	Non-allergen	Non-toxin
3	KWYYVNGLGALAVNT	HLA-DRB1*01:01,HLA-DRB1*09:01,HLA-DRB3*02:02,HLA-DRB1*13:02,HLA-DPA1*03:01/DPB1*04:02,HLA-DRB5*01:01,HLA-DRB1*12:01,HLA-DQA1*05:01/DQB1*03:01,HLA-DRB1*04:05,HLA-DRB1*07:01,HLA-DRB1*04:01,HLA-DRB1*11:01,HLA-DPA1*01:03/DPB1*02:01,HLA-DQA1*01:02/DQB1*06:02	0.433	Non-allergen	Non-toxin
4	GSWYYLNANGAMATG	HLA-DRB1*01:01,HLA-DRB3*02:02,HLA-DRB1*13:02,HLA-DRB1*09:01,HLA-DRB1*04:01,HLA-DRB1*11:01,HLA-DRB3*01:01,HLA-DRB5*01:01,HLA-DQA1*05:01/DQB1*03:01,HLA-DRB1*04:05,HLA-DQA1*01:02/DQB1*06:02,HLA-DRB1*07:01,HLA-DRB1*15:01	0.6041	Non-allergen	Non-toxin
5	GSWYYLNSNGAMATG	HLA-DRB3*02:02,HLA-DRB1*01:01,HLA-DRB1*04:01,HLA-DRB1*09:01,HLA-DRB1*13:02,HLA-DRB1*11:01,HLA-DRB3*01:01,HLA-DRB1*04:05,HLA-DRB5*01:01,HLA-DRB1*07:01,HLA-DQA1*05:01/DQB1*03:01,HLA-DQA1*01:02/DQB1*06:02,HLA-DRB1*15:01	0.5133	Non-allergen	Non-toxin
6	SWYYLNSNGAMATGW	HLA-DRB3*02:02,HLA-DRB1*01:01,HLA-DRB1*13:02,HLA-DRB1*09:01,HLA-DRB1*04:01,HLA-DRB1*11:01,HLA-DRB3*01:01,HLA-DQA1*05:01/DQB1*03:01,HLA-DQA1*01:02/DQB1*06:02,HLA-DRB5*01:01,HLA-DRB1*07:01,HLA-DRB1*04:05	0.516	Non-allergen	Non-toxin
7	WYYLNASGAMATGWA	HLA-DRB1*01:01,HLA-DRB3*02:02,HLA-DRB1*09:01,HLA-DQA1*05:01/DQB1*03:01,HLA-DQA1*01:02/DQB1*06:02,HLA-DRB1*11:01,HLA-DRB1*07:01,HLA-DRB1*13:02,HLA-DRB3*01:01,HLA-DRB1*04:01,HLA-DRB5*01:01,HLA-DRB1*04:05	0.668	Non-allergen	Non-toxin
8	WYYLNANGAMATGWL	HLA-DRB1*01:01,HLA-DRB3*02:02,HLA-DRB1*13:02,HLA-DRB1*09:01,HLA-DQA1*05:01/DQB1*03:01,HLA-DRB1*04:01,HLA-DQA1*01:02/DQB1*06:02,HLA-DRB1*11:01,HLA-DRB5*01:01,HLA-DRB3*01:01,HLA-DRB1*07:01	0.5875	Non-allergen	Non-toxin
9	WYYLNSNGAMATGWL	HLA-DRB3*02:02,HLA-DRB1*01:01,HLA-DRB1*13:02,HLA-DRB1*09:01,HLA-DRB1*04:01,HLA-DRB1*11:01,HLA-DQA1*05:01/DQB1*03:01,HLA-DQA1*01:02/DQB1*06:02,HLA-DRB3*01:01,HLA-DRB5*01:01,HLA-DRB1*07:01	0.4967	Non-allergen	Non-toxin
10	TWYYLEASGAMKASQ	HLA-DRB1*01:01,HLA-DRB5*01:01,HLA-DRB1*09:01,HLA-DRB3*01:01,HLA-DRB1*07:01,HLA-DRB1*04:01,HLA-DQA1*01:02/DQB1*06:02,HLA-DQA1*05:01/DQB1*03:01,HLA-DRB1*13:02,HLA-DRB1*11:01,HLA-DRB1*04:05	0.734	Non-allergen	Non-toxin
11	VNGSWYYLNASGAMA	HLA-DRB1*09:01,HLA-DRB1*09:01,HLA-DRB3*02:02,HLA-DRB3*01:01,HLA-DRB1*07:01,HLA-DRB1*11:01,HLA-DRB1*13:02,HLA-DRB1*04:01,HLA-DRB5*01:01,HLA-DRB1*04:05,HLA-DQA1*05:01/DQB1*03:01	0.5358	Non-allergen	Non-toxin
12	TLIIKLSAIKTEYLR	HLA-DRB1*01:01,HLA-DRB4*01:01,HLA-DRB1*12:01,HLA-DRB1*09:01,HLA-DRB1*15:01,HLA-DRB5*01:01,HLA-DRB1*04:05,HLA-DRB1*13:02,HLA-DRB1*07:01,HLA-DRB1*08:02,HLA-DPA1*03:01/DPB1*04:02,HLA-DPA1*02:01/DPB1*05:01,HLA-DRB1*11:01,HLA-DRB1*04:01	0.5855	Non-allergen	Non-toxin
13	LIIKLSAIKTEYLRE	HLA-DRB1*01:01,HLA-DRB4*01:01,HLA-DRB1*12:01,HLA-DRB1*09:01,HLA-DRB1*04:05,HLA-DRB1*15:01,HLA-DRB5*01:01,HLA-DRB1*13:02,HLA-DRB1*07:01,HLA-DPA1*03:01/DPB1*04:02,HLA-DPA1*02:01/DPB1*05:01,HLA-DRB1*08:02,HLA-DPA1*02:01/DPB1*01:01,HLA-DRB1*04:01	0.5094	Non-allergen	Non-toxin
14	IKLSAIKTEYLRELN	HLA-DRB1*01:01,HLA-DRB4*01:01,HLA-DPA1*03:01/DPB1*04:02,HLA-DRB1*04:05,HLA-DRB1*15:01,HLA-DRB1*07:01,HLA-DRB1*09:01,HLA-DPA1*02:01/DPB1*01:01,HLA-DPA1*01:03/DPB1*04:01,HLA-DPA1*02:01/DPB1*05:01,HLA-DPA1*01:03/DPB1*02:01	0.7839	Non-allergen	Non-toxin
15	KLSAIKTEYLRELNV	HLA-DRB1*01:01,HLA-DPA1*03:01/DPB1*04:02,HLA-DRB4*01:01,HLA-DRB1*15:01,HLA-DRB1*07:01,HLA-DRB1*09:01,HLA-DPA1*01:03/DPB1*04:01,HLA-DPA1*02:01/DPB1*01:01,HLA-DPA1*01:03/DPB1*02:01,HLA-DPA1*02:01/DPB1*05:01	0.7314	Non-allergen	Non-toxin

### Population coverage

**Fig. 3:**
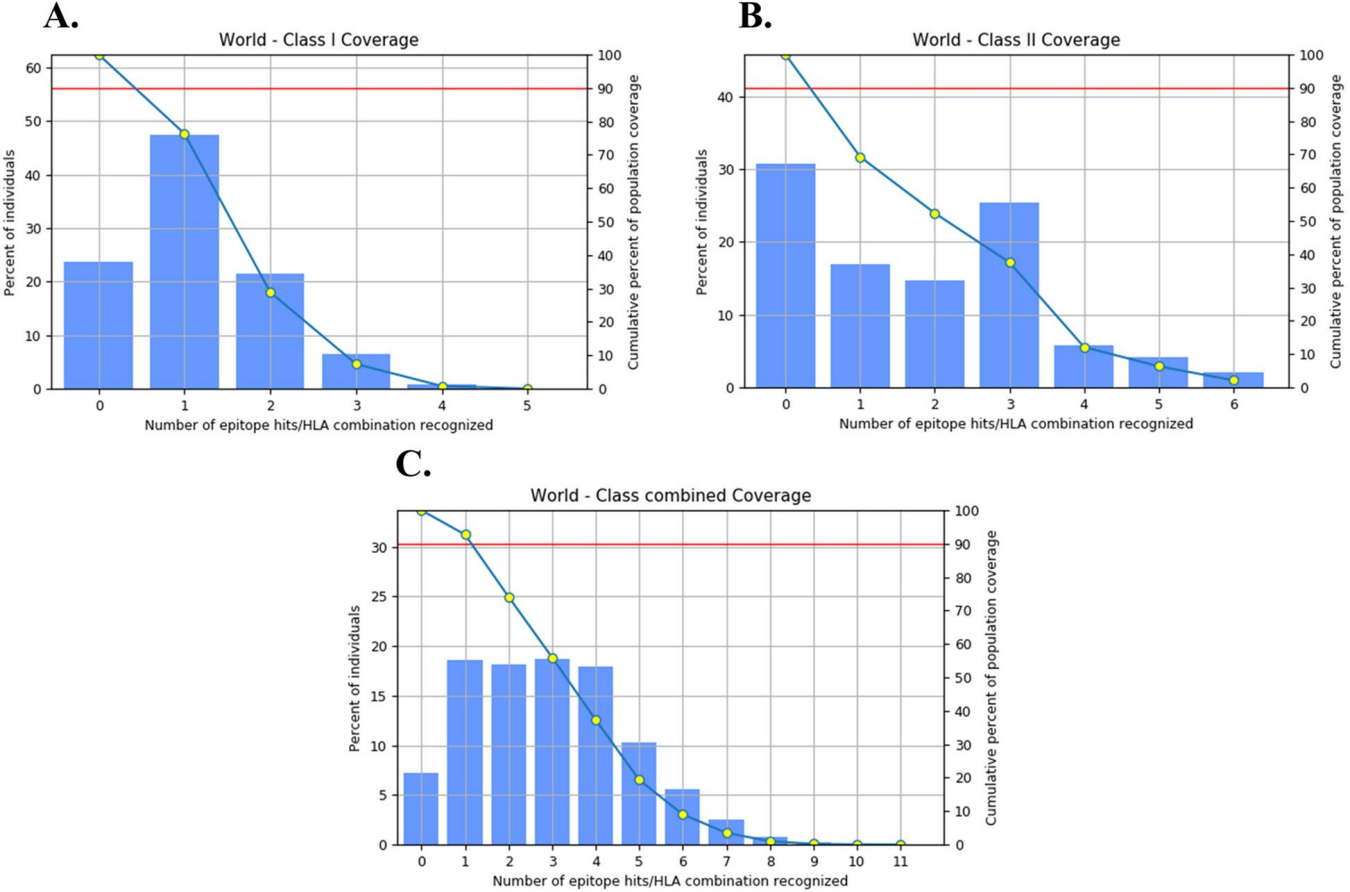
The coverage of the target alleles with the potential epitopes was evaluated for the population. The figure depicts the extent of coverage for MHC-I epitopes (**A**), MHC-II epitopes (**B**), and both types of epitopes together (**C**)

Using the IEDB tool, the coverage of MHC-I and MHC-II alleles with different epitopes was assessed. The analysis revealed that the chosen MHC-I epitopes had a population coverage of 76.34%, while the chosen MHC-II epitopes covered 69.26%. After a comprehensive evaluation of every epitope, the total coverage was an impressive 92.73%, as illustrated in Fig. 3. These results suggest that the chosen epitopes for the vaccine have the potential to be effective worldwide, with only minor variations observed in different ethnic groups.

### Construction of the vaccine

We opted to use a combination of 12 B cell epitopes, 9 MHC-I epitopes, and 15 MHC-II epitopes to build the MEV. To increase the vaccine’s effectiveness, we also employed linkers (EAAAK, CPGPG, and AAY) to join the adjuvant to the B cell epitopes, the B cell epitopes to the MHC-I, and the MHC-II epitopes to each other. Moreover, a 6 × His tag was included in the vaccine's sequence to aid in protein purification and characterization. The vaccine’s structure is depicted in the following Table 4.

**Table 4:** Constructed multi-epitope-based vaccine

Vaccine Construct	
Adjuvant	50S ribosomal protein
EAAK	EAAAK
B cell epitopes	WYYLNA
	LPSPSLKLGKKVAEAE
	WYYLEAS
	NKKLQLD
	AELPSKIKAKLDAAFDQ
	EQPQPAPAPQP
	ERKVHYSIRKFSVGVASVVVASLVMGSVVHAT
	SWYYLNA
	SWYYLN
	SWYYLNS
	QWFKVSDKWYYVNGLGALAVNTT
	LRELNVLED
Linker	CPGPG
MHC-I epitopes	KVSDKWYYV
	RNYPTNTYK
	TLIIKLSAI
	SSNMAKTEYR
	SNMAKTEYR
	NANGAMATGW
	QVATSSNMAK
	LIIKLSAIK
	TLIIKLSAIK
Linker	AAY
MHC-II epitopes	SWYYLNANGAMATGW
	SWYYLNASGAMATGW
	KWYYVNGLGALAVNT
	GSWYYLNANGAMATG
	GSWYYLNSNGAMATG
	SWYYLNSNGAMATGW
	WYYLNASGAMATGWA
	WYYLNANGAMATGWL
	WYYLNSNGAMATGWL
	TWYYLEASGAMKASQ
	VNGSWYYLNASGAMA
	TLIIKLSAIKTEYLR
	LIIKLSAIKTEYLRE
	IKLSAIKTEYLRELN
	KLSAIKTEYLRELNV
6X Histadine Tag	HHHHHH

 > vaccine protein.

EAAAKWYYLNALPSPSLKLGKKVAEAEWYYLEASNKKLQLDAELPSKIKAKLDAAFDQEQPQPAPAPQPERKVHYSIRKFSVGVASVVVASLVMGSVVHATSWYYLNASWYYLNSWYYLNSQWFKVSDKWYYVNGLGALAVNTTLRELNVLEDCPGPGKVSDKWYYVRNYPTNTYKTLIIKLSAISSNMAKTEYRSNMAKTEYRNANGAMATGWQVATSSNMAKLIIKLSAIKTLIIKLSAIKAAYSWYYLNANGAMATGWSWYYLNASGAMATGWKWYYVNGLGALAVNTGSWYYLNANGAMATGGSWYYLNSNGAMATGSWYYLNSNGAMATGWWYYLNASGAMATGWAWYYLNANGAMATGWLWYYLNSNGAMATGWLTWYYLEASGAMKASQVNGSWYYLNASGAMATLIIKLSAIKTEYLRLIIKLSAIKTEYLREIKLSAIKTEYLRELNKLSAIKTEYLRELNVHHHHHH.

### Analysis of physicochemical and immunological properties

The vaccine construct consists of 438 building blocks, and the computed molecular weight was 49,134.28. The predicted isoelectric point (PI) of the protein is 9.51, indicating a positive charge, as isoelectric points above 7.0 are positively charged. The protein was classified as stable with an instability index (II) of 25.61, as determined by Protparam. The aliphatic index is 82.62, meaning that it can withstand a wide range of temperatures. The calculated grand average of hydropathicity (GRAVY) using the chemical formula C_2482_H_3708_N_626_O_684_S_16_ was -0.170. According to the Vexijen v2.0 server, the vaccine sequence we created is likely an antigen with an antigenic score of 0.6872. Afterward, Allertop v2.0 verifies that the structure is not an allergen, while Toxinpred confirms that it is not toxic.

### Predicting, improving and verifying the 3D structure of vaccine

**Fig. 4:**
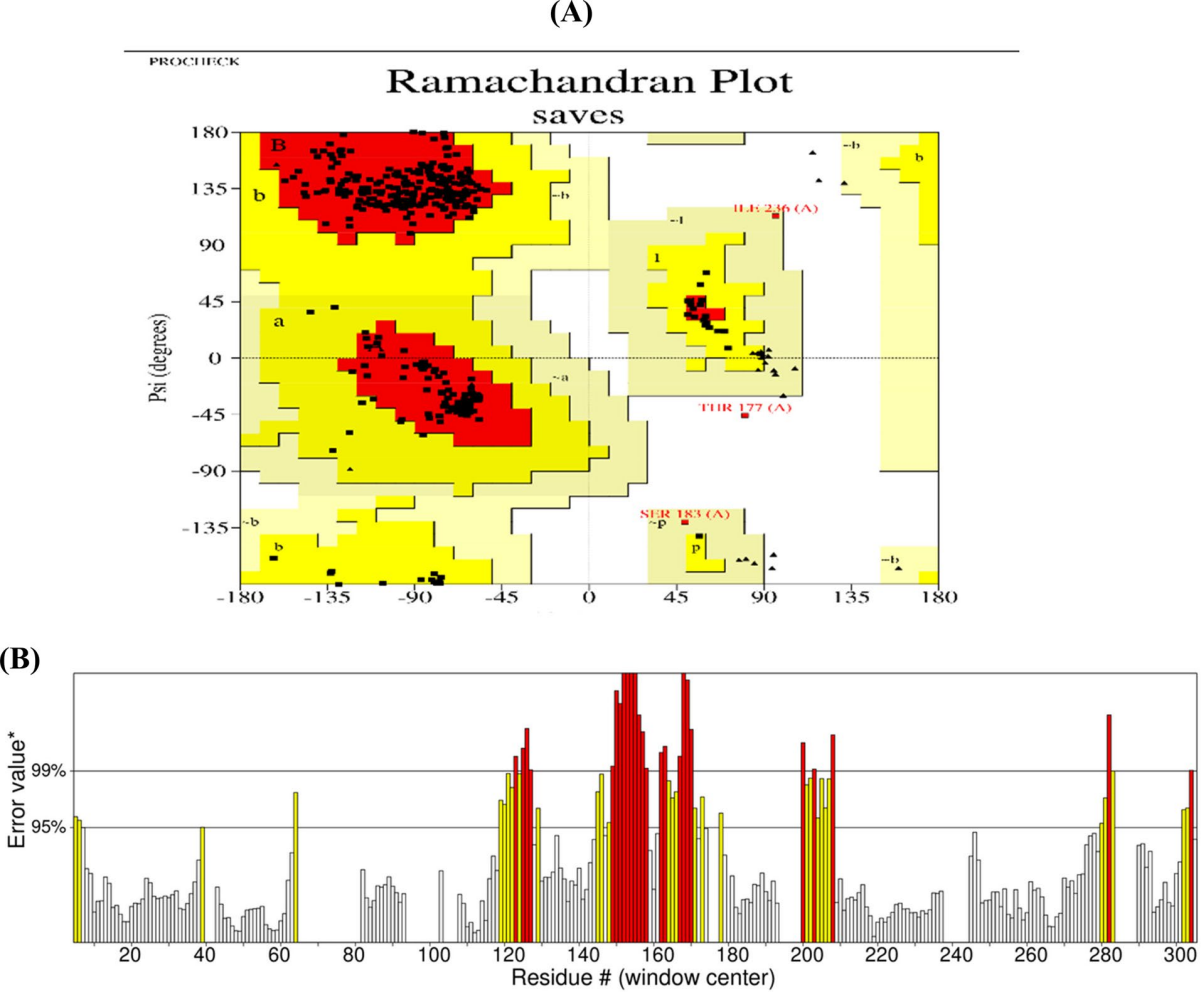
Tertiary structural verification of the vaccine construct. The Ramachandran plot of the modified model (**A**) indicates that 91.4% of the 3D residues are situated within the optimal region, while (**B**) represents an assessment of the improved model’s ERRAT quality factor (84.53)

**Fig. 5:**
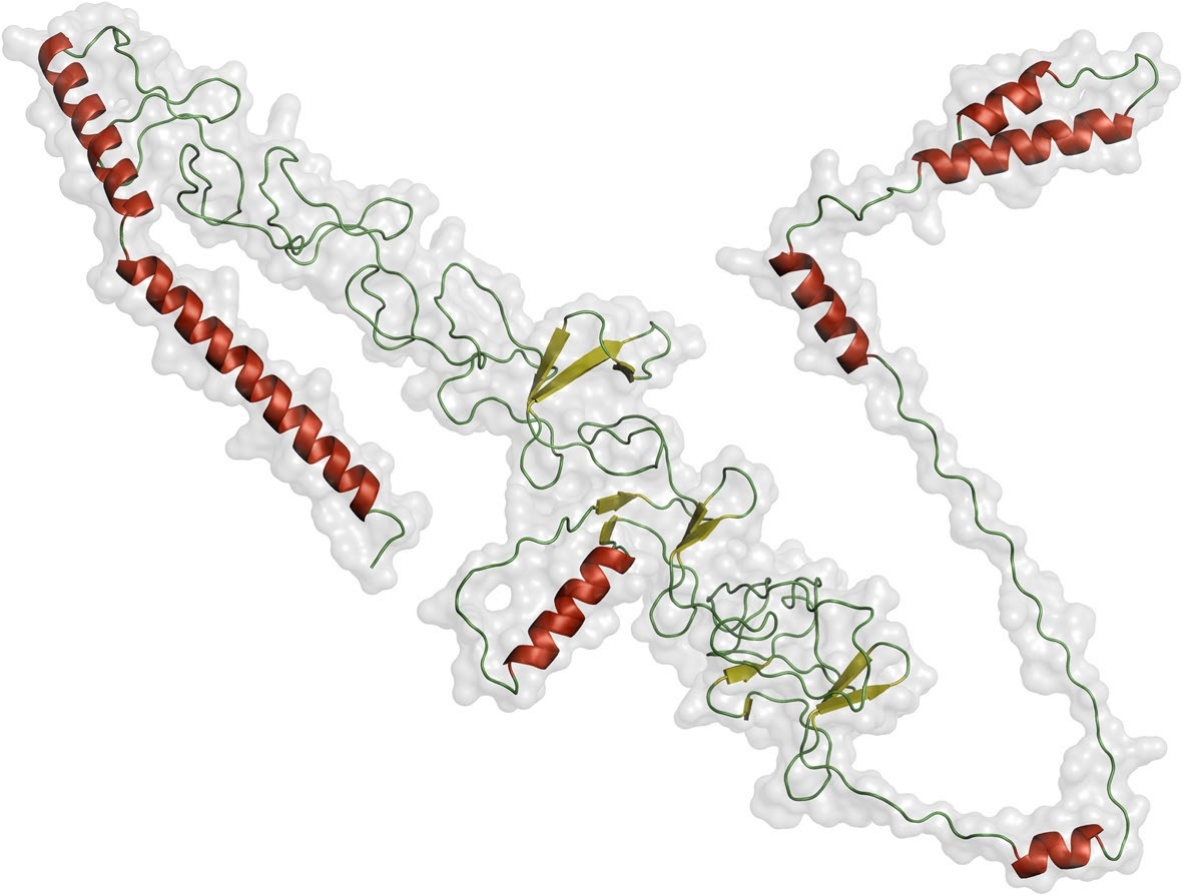
Rendering of vaccine’s 3D structure in PyMol 2

The trRosetta server created five different 3-dimensional structures of the intended vaccination sequence, and the best one was selected. The protein structure was stabilized and given a higher quality score on the SAVES server after being refined by the Galaxy server. Analyzing the Ramachandran plot for the revised structure (Fig. 4A) showed that 91.4% of the 3D residues were in the optimal area. Similar patterns can be seen in the ERRAT program, where the total quality factor was raised to 84.53 (Fig. 4B). PyMol 2 was used to generate the 3D representation of the final vaccine structure shown in Fig. 5.

### Molecular docking of the TLR4 receptor-vaccine construct

**Fig. 6:**
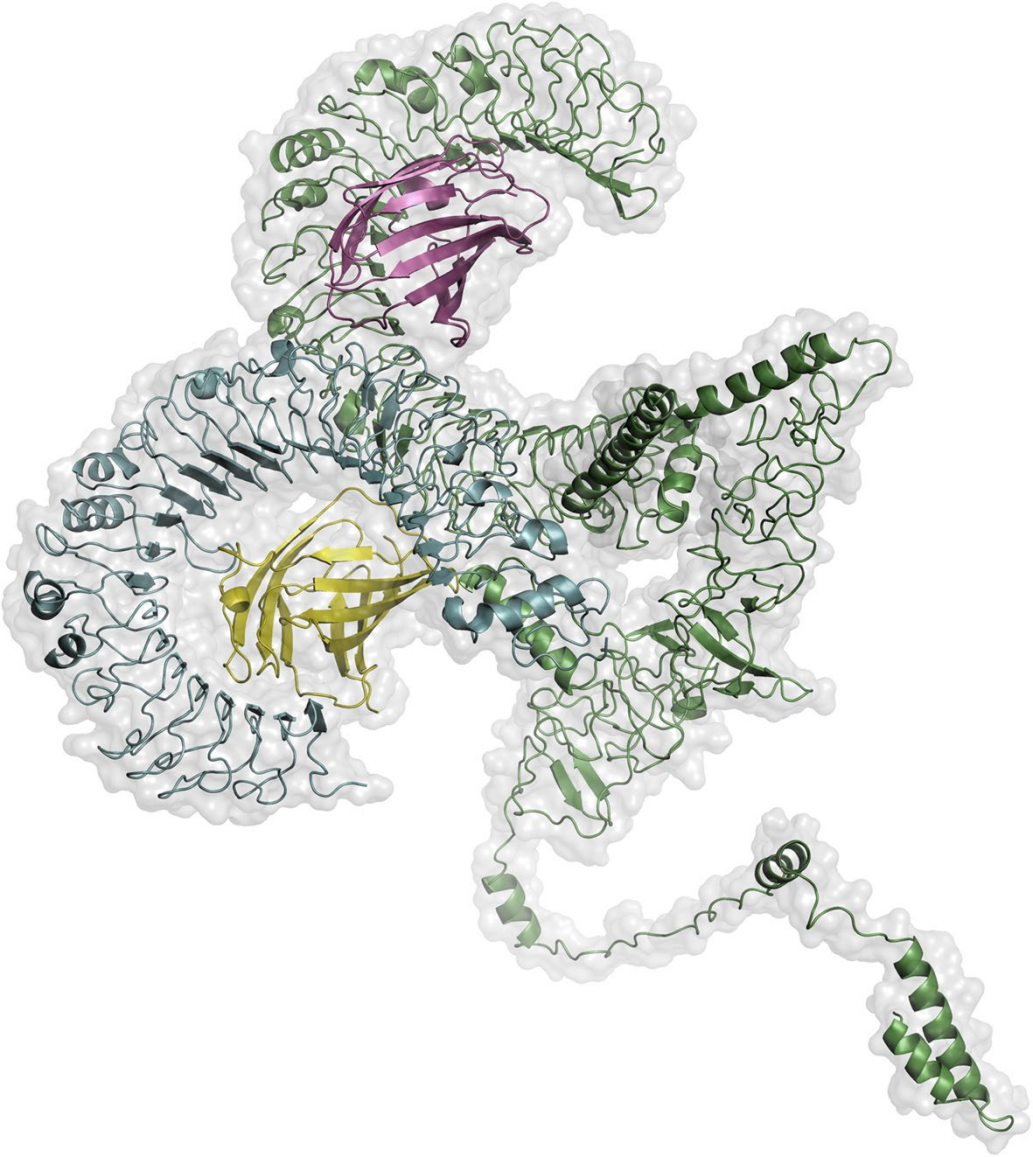
PyMol visualization of cluspro-derived docked complex with 32 clusters and a minimum energy score of − 1133.3

Ten different models were analyzed using Cluspro docking analysis predictions. After a visual comparison of all ten docking models using Pymol, the model with the least amount of energy consumption and the highest number of contributors to the cluster’s formation was selected (depicted in Fig. 6). This model produced a satisfactory docking result, with a binding score of − 1133.3 over 32 clusters.

### Molecular dynamics simulation

**Fig. 7:**
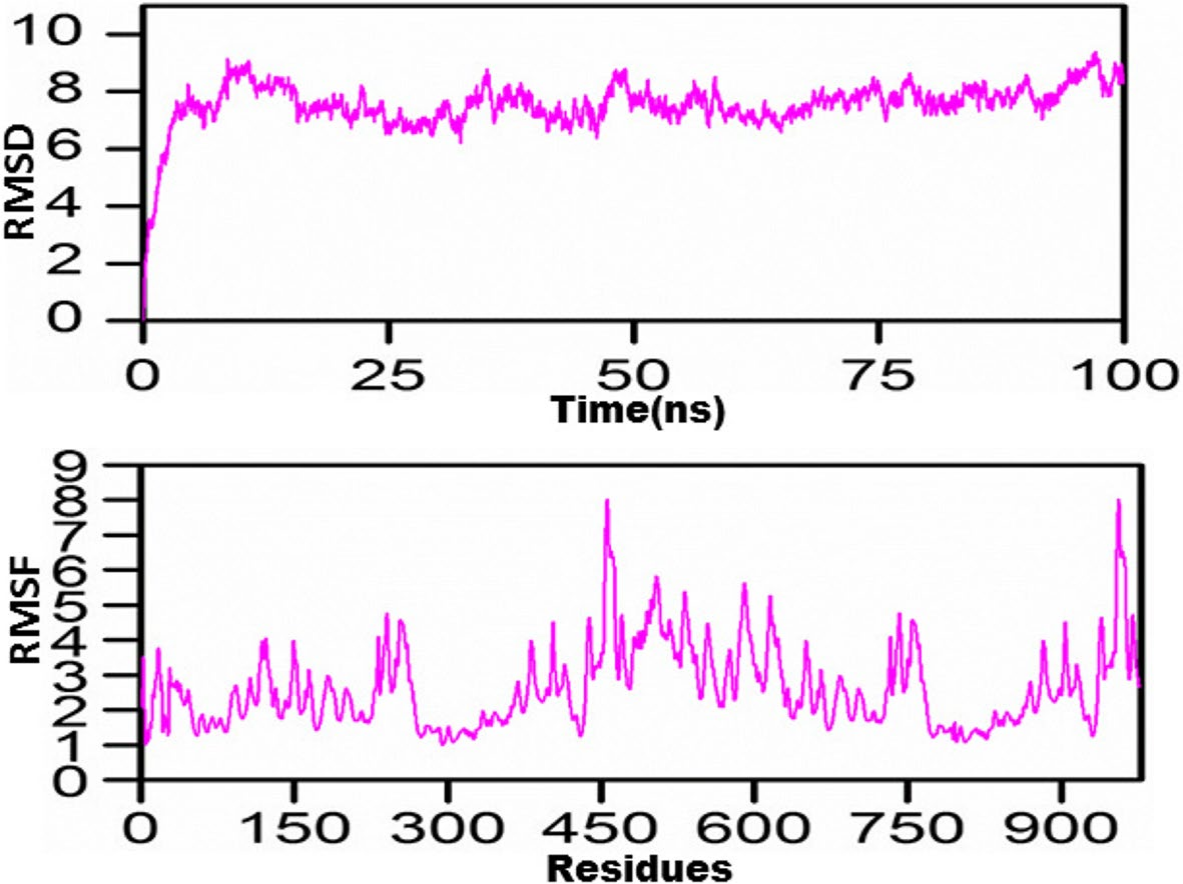
MD simulation findings of the vaccine construct for 100 ns. RMSD (**A**) and RMSF (**B**) were elucidated

The MD simulations performed for the constructed vaccine revealed its stable nature, as reflected by the RMSD diagram since this stability is achieved in from the first 10 ns. This emphasized the folding stability as well as the almost sustained behavior of the vaccine construct. Furthermore, the residual fluctuations were recorded to detect the RMSF, which was found to be minimal; however, little fluctuation was recorded between 75 and 100 ns. In addition, marked RMSF was seen at the end, which may account for the highly flexible loops at both terminals (N-terminal and C-terminal) (Fig. 7A,B).

### Simulation of the immune system

**Fig. 8:**
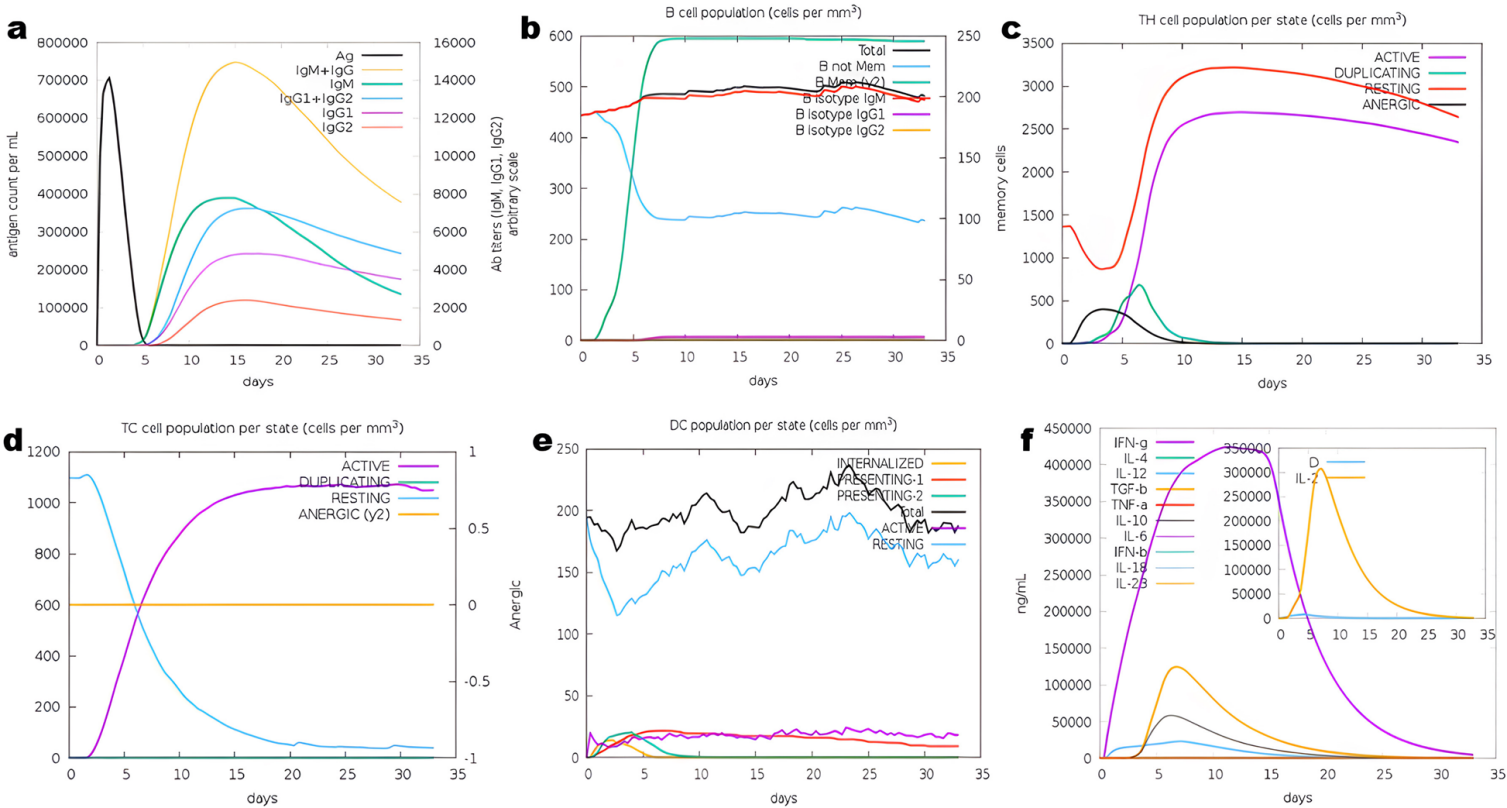
Vaccine immune simulation through the C-ImmSim server

Using the C-ImmSim website, we assessed the vaccine’s capacity to produce an effective immunological response in real-world conditions. The subsequent and tertiary immune responses increased progressively after the first response. Figure 8 shows the large rise in antibody levels (IgM, IgG + IgM, and IgG1 + IgG2), and the pattern of the immune reaction was comparable to that of usual human immunological responses. Figure 8a displays the presence of IgM and IgG antibodies and the formation of memory cells. Figure 8b shows a substantial increase in the number of B-cells, with both IgG1 and IgM biotypes present, and a significant increase in memory cell formation. While the number of activated T cells showed a sharp increase after the third and fourth injections, they gradually decreased at later stages, as evident in Fig. 8c, d. Furthermore, both Fig. 8e, f demonstrated an increase in the number of TH cells and IFN- levels.

### Optimization of codons

**Fig. 9:**
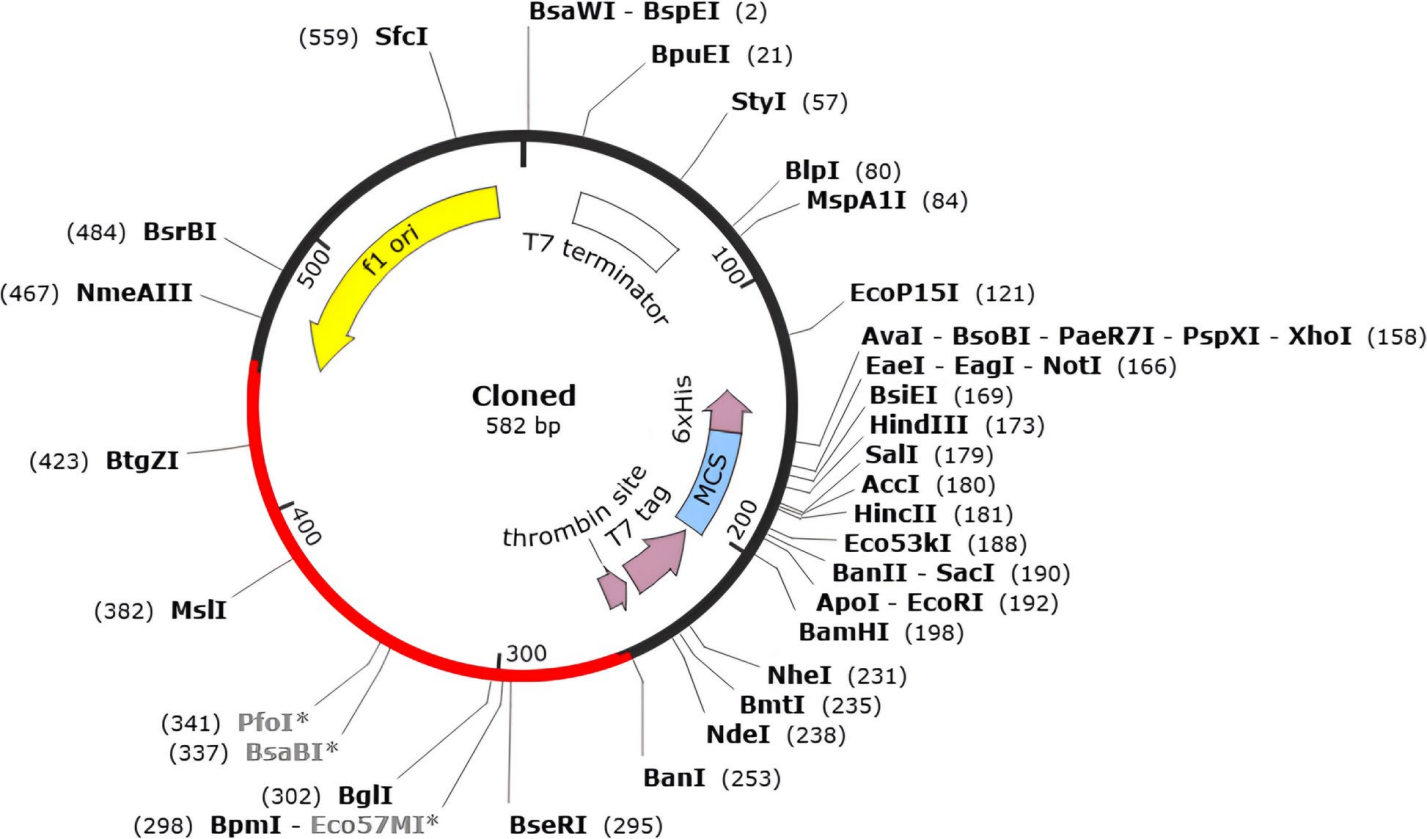
The final MEV was in silico cloned using pET28a ( +). The vector is depicted by a black circle, while the insertion location for the vaccination is shown by the red area

We utilized the Jcat tool to determine codon optimization and reverse translation in *E. coli*, resulting in a high expression of the vaccine. In all, there are 720 bases in the codon-optimized sequence. The GC percentage of the cDNA sequence was determined to be 64.42%, which is in the optimal range of 30–70%. Codon optimization assesses the sequence and provides information on the codon adaptive index (CAI) and GC content of the cDNA sequence. The CAI value, which was computed at 0.95 and also falls within the range of (0.8–1.0), suggests that the vaccine candidate may express well in the *E. coli* host. Following the development of BanI and TaiI restriction sites, the vector pET28a ( +) was cloned using SnapGene software (Fig. 9). Thus, the clone’s total length was 582 bp.

## Discussion

The most prevalent bacterial organism connected to bacterial pneumonia is *Streptococcus pneumoniae*. The burden of illness falls on underdeveloped countries because of inadequate immunization programs [60]. The NIH reports that *S. pneumoniae* remains a significant source of morbidity and mortality worldwide, particularly among children and the elderly, and is categorized as a high-burden disease [61]. Although currently available pneumococcal vaccines, such as PPV and PCV, can prevent various types of pneumococcal disease, they have been known to fail in some cases due to serotype replacement [62]. The production complications and high costs associated with PCVs have rendered them unaffordable, particularly in developing nations [63]. Furthermore, there has been an increase in antibiotic resistance among the serotype replacement strains. However, immunoinformatic approaches can be used to address these issues. Using in silico methods can reduce the amount of time and money required for experiments. Immunoinformatics-assisted epitope identification has multiple applications in epitope mapping, including advancements in peptide-based vaccine research, characterization of immunological processes, and prediction of epitopes used in the diagnosis of disease [64]. Epitope-based vaccines are an attractive and prospective new method for developing vaccines, as they employ only fragments of peptides that are known to be highly immunogenic and capable of evoking immune responses [65]. Therefore, our approach aimed to identify a potential epitope-based pneumococcal vaccine candidate that would ideally be conserved across most serotypes, have broad population coverage, and induce T cell-dependent immune responses.

Vaccines based on epitopes must contain B and T cell epitopes that induce potent immune responses against a specific infection [66]. Traditional wet-lab approaches for identifying potential B and T cell epitopes involve experimental screening of numerous active and inactive epitopes, which can be time-consuming and costly. As an alternative, computational methods offer a cost-effective, rapid, reliable, and accurate approach [67, 68]. In this regard, the use of a consensus prediction strategy is proven to be more reliable and robust compared to individual prediction methods [69]. In order to construct a MEV, first B and T-cell epitopes were anticipated using reliable databases. Then the vaccine formulation included linkers such as EAAAK, AAY, and CPGPG, which facilitated better and longer-lasting protection. A significant challenge with epitope vaccines is their vulnerability to degradation by proteases in the body [70]. To address this issue, the vaccine sequences were inserted into the 50S ribosomal chromosome as an adjuvant. According to computational analysis, the manufactured vaccine has been shown to be non-allergenic and highly antigenic (0.68). The proposed vaccine has a high probability of being thermostable, as estimated by the aliphatic index formula [71]. The proposed vaccine exhibits hydrophilicity and preferred interactions with water molecules, as shown by its GRAVY index of − 0.162 [72]. The designed vaccine is stable, as shown by its instability index of 25.62, which is lower than 40. The utilization of a 3D structural model provides valuable insights into protein dynamics, ligand interactions, function, and spatial organization. Substantial refinement of the vaccine's formulation has notably improved its desired properties. The majority of the residues were found to be within the permissible range when the vaccine design was examined using a Ramachandran plot. This indicates that the design of the vaccine is of high quality. The subsequent critical step in validating a vaccine involves molecular docking. In order to generate a powerful immune response in the host, a low binding energy score between the receptor and ligand is required. The docking studies conducted in this investigation revealed a significantly reduced binding energy of − 1133.3 kcal/mol, indicating a strong interaction between the manufactured vaccine and the TLR4 receptor molecule. We also conducted MD simulations, and the analysis showed our vaccine maintains its structural stability. The immune simulation graph demonstrates a notable increase in IgM production following the administration of our designed vaccine, indicating the occurrence of a primary immune response. Additionally, the enhanced expression of immunoglobulins in B cells corresponded to a reduction in antigen concentration. JCat was utilized to modify the codons of the vaccine to improve its expression in *E. coli*. The vaccine’s construction has a CAI value of 0.95 and a GC content of 64.42%. Our findings are excellent since CAI values over 0.8 and GC contents between 30 and 70% are thought to be good for expression. The purpose of the *Escherichia coli* in silico cloning was to lay the groundwork for later wet laboratory studies by other researchers attempting to build an effective vaccine. However, more validation via in vitro and in vivo experiments using animal models is required to confirm the effectiveness of the developed MEV.

## Conclusion

In this current study, we have developed a vaccine candidate against *Streptococcus pneumoniae* using immunoinformatic techniques. We used various online tools and databases to select suitable proteins, predict B and T cell epitopes to construct vaccine candidates and evaluate their physicochemical properties, antigenicity, allergenicity, and stability. In order to evaluate the vaccine’s interaction and binding affinity, we also ran molecular docking and molecular dynamics simulations with human toll-like receptor 4 (TLR-4). Our results suggested that our vaccine candidate has high immunogenicity, broad population coverage, and a low risk of adverse effects. It also showed a strong and stable interaction with human TLR-4, indicating the potential to elicit both adaptive and innate immunity*.* However, these results are based on computational predictions and need to be validated experimentally. Therefore, we recommend further testing of our vaccine candidate in appropriate tissue culture and animal models to confirm its efficacy and safety before proceeding to clinical trials.

## Data Availability

The datasets supporting the conclusions of this article are included within the article.

## Abbreviations

MHC: Major histocompatibility complex
TLR: Toll-like receptor
NCBI: National Center for Biotechnology Information
IEDB: Immune Epitope Database
CAI: Codon Adaptation Index
GC: Guanine-cytosine
